# Mr. Ring, on the Case at Kensington

**Published:** 1804-11-01

**Authors:** John Ring

**Affiliations:** New Street, Hanover Square


### *To the Editors of the Medical and Physical Journal.*

Gentlemen,

In your last number I gave some account of a case which lately occurred at Kensington. I there intimated, that Mr. Merriman of that place, who, it was pretended, could attest the facts, was not sufficiently acquainted with the case to be a witness. It has been represented to me, that the concise manner in which I expressed my sentiments, might gave rise to misconceptions: I therefore requested Mr. Merriman to favour me with an account of what he knows of the case, that I might insert it in your Journal. In consequence of this application, I received the following statement.

"I have not seen the Review; nor did I ever hear that any notice had been taken of the case; much less that my name had been made use of in so unwarrantable a manner; or I should have insisted on the Editors of that publication contradicting that part of the account, which relates to my attesting the fact of the child having had the cow-pock in a regular manner. My attestation can go no farther than as to the small-pox."

I have also received a statement from Mr. Cockle and Mr. Faithorne; in which they declare, that *when particularly questioned* concerning the scab on the child's arm, after the pretended cow-pock, Mr. Meredith *positively affirmed it never turned black, nor even dark.*

In one respect, the Kensington case resembles that in Fullwood's Rents. It is easy to prove the child had the small-pox: the only difficulty is, to prove that it ever had the genuine cow-pock.

I am, &c.

*New Street, Hanover Square.* JOHN RING.

